# Variation in Retinal Nerve Fiber Layer and Ganglion Cell Complex Associated With Optic Nerve Head Size in Healthy Eyes

**DOI:** 10.1167/tvst.12.3.26

**Published:** 2023-03-27

**Authors:** Caixia Li, Yanyan Cheng, Ye Zhang, Xiaohua Pan, Hui Feng, Fei Xiang, Meijuan Zhang, Qianqian Ji, Zhi Li, Na Jiang, Qing Zhang, Shuning Li

**Affiliations:** 1 School of Clinical Medicine, Dali University, Dali, China; 2 Hebei Eye Hospital, Hebei Provincial Key Laboratory of Ophthalmology, Hebei Provincial Clinical Research Center for Eye Diseases, Xingtai, China; 3 Beijing Tongren Eye Center, Beijing Tongren Hospital, Beijing Ophthalmology & Visual Sciences Key Laboratory, Capital Medical University, Beijing, China; 4 Shenzhen Eye Hospital, Jinan University, Shenzhen Eye Institute, Shenzhen, China; 5 Beijing Institute of Ophthalmology, Beijing Tongren Hospital, Capital University of Medical Science, Beijing Ophthalmology and Visual Sciences Key Laboratory, Beijing, China

**Keywords:** retinal nerve fiber layer, ganglion cell complex, optic nerve head

## Abstract

**Purpose:**

To investigate whether the retinal nerve fiber layer (RNFL) and ganglion cell complex (GCC) change with optic nerve head (ONH) size in healthy eyes.

**Methods:**

This cross-sectional observational study recruited participants aged ≥50 years. Participants underwent optical coherence tomography–assisted measurements of the peripapillary RNFL and macular GCC and were divided into small, medium, and large ONH groups according to optic disc area (≤1.9 mm^2^, >1.9 mm^2^ and ≤2.4 mm^2^, and >2.4 mm^2^, respectively). The groups were compared for RNFL and GCC. Linear regression models were used to evaluate the correlation of RNFL and GCC with ocular and systemic factors.

**Results:**

There were 366 participants. The whole, temporal, and superior RNFLs were significantly different among the groups (*P* = 0.035, 0.034, and 0.013, respectively) with no significant difference in the nasal and inferior RNFL (*P* = 0.214, 0.267, respectively). The average, superior, and inferior GCCs were not significantly different among the groups (*P* = 0.583, 0.467, and 0.820, respectively). Thinner RNFL was independently associated with older age (*P* = 0.003), male sex (*P* = 0.018), smaller disc area (*P* < 0.001), higher vertical cup disc ratio (VCDR) (*P* < 0.001), and larger maximum cup depth (*P* = 0.007); thinner GCC was independently associated with older age (*P* = 0.018), larger best-corrected visual acuity (*P* = 0.023), and higher VCDR (*P* = 0.002).

**Conclusions:**

RNFL but not GCC significantly increased with ONH size in healthy eyes. GCC may be more suitable than RNFL for evaluating early glaucoma in patients with large or small ONH.

**Translational Relevance:**

GCC may be a better index than RNFL for early glaucoma evaluation in patients with large or small ONH.

## Introduction

Glaucoma is the leading cause of irreversible blindness worldwide. The global prevalence of glaucoma is approximately 3.54% for people aged 40 to 80 years, and it is anticipated that 111.8 million people worldwide will be affected by the disease by 2040.^1^ Morphologic changes in glaucoma precede functional changes detected in the visual field.^2^^–^^4^ Previously, only peripapillary retinal nerve fiber layer (RNFL) measurements were widely used to assess structural changes in early glaucoma.^5^^,^^6^ However, some experimental glaucoma studies in monkeys have found a large loss of retinal ganglion cells (RGCs) in the macular region.^7^^,^^8^ A large part of RGCs are located in the macular region, which makes this area important for glaucoma investigation.^9^ We postulate that the evaluation of glaucoma should focus not only on the loss of peripapillary RNFL thickness but also on the loss of macular ganglion cell complex (GCC) thickness.

In recent years, research in the field of glaucoma has focused on identifying a more accurate and reliable early glaucoma detection parameter. At present, peripapillary RNFL and macular GCC thickness measurements are the most widely used clinical parameters for early glaucoma diagnosis and follow-up, and these are strongly correlated.^10^^–^^12^ Previous studies have demonstrated that RNFL thickness measurements are affected by numerous factors, particularly optic nerve head (ONH) size,^13^ axial length (AL), and magnification.^14^ ONH size is not constant among individuals but shows interindividual variability.^15^ Several studies have demonstrated a correlation between RNFL thickness and ONH size, although results were inconsistent. For example, a number of studies suggest that RNFL thickness increases with an increase in ONH size.^13^^,^^16^^–^^18^ However, some researchers have found no significant association between RNFL thickness and ONH size and maintain that AL affects the magnification of the fundus image.^19^ Furthermore, only few previous studies have investigated the relationship between GCC thickness and ONH size, with conflicting conclusions. Rao et al.^20^ evaluated the impact of ONH size on the diagnostic accuracy of GCC thickness in glaucoma diagnosis and found that GCC thickness was not affected by ONH size. Conversely, another study reported a significant positive correlation between ONH size and macular GCC, suggesting that ONH size is an important influence on macular GCC thickness.^21^ In addition, Cordeiro et al.^22^ divided the area of the optic disc into 1.5 mm^2^, 2.0 mm^2^, and 2.5 mm^2^ and compared the predicted areas and sensitivity under receiver operating characteristic curves of RNFL and GCC for each fixed area of the optic disc. The results showed that the diagnostic accuracy of RNFL and GCC thickness parameters was similar. However, RNFL thickness measurements were a better diagnostic predictor in small discs, whereas GCC measurements were a better diagnostic predictor in large discs. This study aimed to investigate the associations between peripapillary RNFL and macular GCC thickness and ONH size and to identify more reliable indicators for the detection and diagnosis of early glaucoma.

## Material and Methods

### Patients

This cross-sectional, observational study was conducted between October 2018 and November 2018 in the Daxing District, Beijing. A total of 366 eyes of 366 participants (93 men and 273 women) aged ≥50 years were included in this study. The study was approved by the Ethics Committee of Clinical Research at Beijing Tongren Hospital, Capital Medical University. The protocol was in accordance with the principles of the Declaration of Helsinki. Written informed consent was obtained from all participants. This study was registered in the Chinese Clinical Trial Registry (registration number: ChiCTR1900022276).

### Clinical Examinations

All participants underwent systemic and comprehensive ophthalmic examinations, and the following data were collected: age, sex, height, weight, measurement of best-corrected visual acuity (BCVA), spherical refractive error (SER), intraocular pressure (IOP), AL, disc area, cup area, vertical cup disc ratio (VCDR), maximum cup depth (MCD), and RNFL and GCC thickness (Optovue; Optovue, Inc., Fremont, CA, USA). BCVA was measured using Snellen charts and converted to a log scale. After vision and refraction examinations, IOP was measured using a noncontact tonometer without anesthesia (Topcon CT-80; Topcon Medical Systems, Inc., Oakland, NJ, USA). The AL was measured using Lenstar (Lenstar LS900; Haag Streit, Bern, Switzerland). Fundus photography (Kowa Nonmyd WX, Tokyo, Japan) was used to measure the size of the disc area, cup area, VCDR, and MCD. Peripapillary RNFL and macular GCC thicknesses were obtained using 80-kHz RTVue XR optical coherence tomography (OCT) with AngioVue software 2017.1 (Optovue; Optovue, Inc., Fremont, CA, USA). Slit-lamp examination (Haag Streit), binocular optic disc evaluation, and gonioscopy (Ocular Technology, Inc., Goleta, CA, USA) were performed by experienced glaucoma specialists (SNL and YH).

### Inclusion and Exclusion Criteria

The inclusion criteria were as follows: (1) age ≥50 years, (2) IOP ≤21 mm Hg, (3) SER between −6 and +6 diopters, and (4) normal appearance of the ONH, including a VCDR of <0.7 and a difference in VCDR of <0.2 between both eyes. The exclusion criteria were as follows: (1) family history of glaucoma; (2) prior laser, refractive, or intraocular surgery; (3) significant ocular disease such as glaucoma or suspected glaucoma, presence of optic neuropathy or fundus disease, epiretinal membranes, history of intraocular inflammation, trauma, tumors, or optic nerve anomalies; and (4) poor quality of fundus stereographic images and OCT images with an overall quality index <6 or severe artifacts.

### OCT Imaging

The RNFL and GCC parameters were measured using OCT (Optovue; Optovue, Inc., Fremont, CA, USA), and imaging was performed using the tracking mode. An experienced examiner performed all scans. Various RNFL and GCC parameters (whole, temporal, superior, nasal, inferior RNFL; average, superior, and inferior GCC thickness) were measured using the AngioVue software RTVue XR Avanti System Version 2017.1 (Optovue). AngioVue analysis automatically segmented the peripapillary region into eight Garway–Heath segments, including the nasal superior, nasal inferior, inferior nasal, inferior temporal, temporal inferior, temporal superior, superior temporal, and superior nasal. OCT image quality is described by the overall quality index (QI); QI < 6 or severe artifacts were excluded.

### Stereoscopic Fundus Imaging

The optic nerve parameters were measured using a stereoscopic fundus (Kowa Nonmyd WX) without dilation. The field angles with a square mask were 34° (20° horizontal and 27° vertical). Parameters of the optic nerve, including disc area, cup area, VCDR, and MCD, were automatically reported by the built-in software of Kowa Nonmyd WX (KOWA VK-2 WX).

Participants were classified into three ONH size groups depending on the disc area: small-ONH group (disc area ≤1.9 mm^2^), medium-ONH group (disc area >1.9 mm^2^ and ≤2.4 mm^2^), and large-ONH group (disc area >2.4 mm^2^).^23^

### Statistical Methods

All statistical analyses were performed using IBM SPSS Statistics 25.0 program (SPSS, Inc., Chicago, IL, USA). The Kolmogorov–Smirnov and the Shapiro–Wilk tests were used to assess whether continuous variables were normally distributed. Quantitative data were represented as mean and SD (or median, interquartile range), and qualitative data were expressed as percentages. To compare differences between excluded and included groups, independent samples *t*-test or Mann–Whitney *U* test was used for quantitative variables and chi-square test for qualitative variables. Furthermore, the relationships between sectionalized RNFL and GCC thickness and ONH size were determined using analysis of variance or the Kruskal–Wallis test with the Bonferroni correction. Univariate and multivariate regression analyses were performed to identify the associations between RNFL and GCC thickness with other ocular and systemic parameters. The significance level was set at *P* < 0.05.

## Results

### Comparisons of Characteristics Between Excluded and Included Individuals

#### Study Population

The demographic characteristics of the study population are presented in Table 1. A total of 366 eyes of 366 participants were included in the analysis (93 men and 273 women). Participant age ranged from 50 to 82 years, with a median of 60 (55.75–65) years. Compared with the excluded group, the included group tended to be significantly younger (*P* = 0.026), with lower IOP (*P* = 0.006) and thicker RNFL (*P* < 0.001) (Table 1).

**Table 1.** Comparisons of Demographic Characteristics Between Included and Excluded Individuals

Variable	Excluded Group (*n* = 72)	Included Group (*n* = 366)	*P* Value
Age (y)	63 (56–68)	60 (55.75–65)	**0.026**
Sex (men), *n* (%)	19 (26.4)	93 (25.4)	0.862
BMI (kg/m^2^)	27.61 (25.43–29.23)	27.10 (25.03–29.47)	0.738
IOP (mm Hg)	17 (15–19.75)	16 (15–18)	**0.006**
BCVA (logMAR)	0.00 (0.00–0.22)	0.00 (0.00–0.10)	0.160
SER (diopter)	0.44 (–0.50 to 1.59)	0.50 (–0.125 to 1.25)	0.592
AL (mm)	22.79 ± 0.66	22.82 ± 0.82	0.797
Disc area (mm^2^)	2.41 (2.19–2.78)	2.54 (2.21–2.91)	0.118
Cup area (mm^2^)	0.32 (0.24–0.53)	0.34 (0.24–0.50)	0.540
VCDR	0.34 (0.29–0.43)	0.35 (0.30–0.41)	0.782
MCD (mm)	0.27 (0.16–0.36)	0.28 (0.20–0.37)	0.175
RNFL thickness (µm)	107.03 ± 13.10	112.82 ± 12.29	**<0.001**
GCC thickness (µm)	96.53 (89.51–100.63)	96.60 (92.40–100.85)	0.197

Values are presented as mean ± SD or median (interquartile range) unless otherwise indicated. Values with statistical significance are shown in boldface.

#### Associations Between RNFL and GCC Thickness and ONH Size

The distribution of sectionalized RNFL and GCC thickness by ONH size is shown in Table 2. In all ONH size groups, the inferior quadrant RNFL was thicker than the superior quadrant, followed by the nasal and temporal quadrants. Significant differences were observed in the whole, temporal, and superior RNFLs (*P* = 0.035, 0.034, and 0.013, respectively). In addition, the whole, temporal, and superior RNFLs were significantly different between the small- and large-ONH groups (*P* = 0.031, 0.072, and 0.017, respectively) when compared to both control groups after the Bonferroni correction. However, no significant differences were found among the three study groups in the nasal and inferior RNFLs (*P* = 0.214 and *P* = 0.267, respectively) or in the average, superior, and inferior GCCs (*P* = 0.583, *P* = 0.467, and *P* = 0.820, respectively) (Table 2).

**Table 2.** Comparisons of Sectionalized RNFL and GCC Thicknesses Between Different ONH Size Groups

	Optic Disc Area/ONH size						
Segment	Small (*n* = 35)	Medium (*n* = 111)	Large (*n* = 220)	*P* Value	*P*1	*P*2	*P*3
RNFL thickness (µm)							
Whole	108.04 ± 14.22	112.45 ± 10.43	113.76 ± 12.69	**0.035**	0.190	**0.031**	1.000
Temporal	70.92 ± 11.25	73.12 ± 9.07	75.30 ± 11.22	**0.034**	0.857	**0.072**	0.237
Superior	128.10 (113.53–140.99)	134.61 (122.79–143.46)	137.24 (124.81–147.83)	**0.013**	0.298	**0.017**	0.334
Nasal	95.52 ± 18.38	99.17 ± 13.67	100.31 ± 15.29	0.214			
Inferior	142.66 ± 20.61	148.33 ± 18.26	147.79 ± 18.40	0.267			
GCC thickness (µm)							
Average	95.49 ± 6.26	96.85 ± 6.40	96.78 ± 7.56	0.583			
Superior	94.96 ± 6.134	96.72 ± 6.82	96.35 ± 7.80	0.467			
Inferior	97.04 (92.50–100.79)	97.12 (93.67–100.41)	97.21 (92.20–102.02)	0.820			

Values are presented as mean ± SD or median (interquartile range). Values with statistical significance are shown in boldface. *P*1, small versus medium; *P*2, small versus large; *P*3, medium versus large.

#### Distribution of RNFL and GCC Thickness

The mean RNFL thickness in this study was 112.82 ± 12.29 µm. The inferior quadrant of the RNFL was the thickest, followed by the superior, nasal, and temporal quadrants. In addition, the large ONHs, significantly in the superior quadrant, were the thickest among all segments, followed by the medium and small ONHs, as shown in Figure A. The median thickness of the GCC in this study was 96.60 (92.40–100.85) µm. The inferior quadrant of the GCC was the thickest, followed by the average and superior quadrant. However, there was no significant difference between the different ONH groups, as shown in Figure B.

**Figure.**
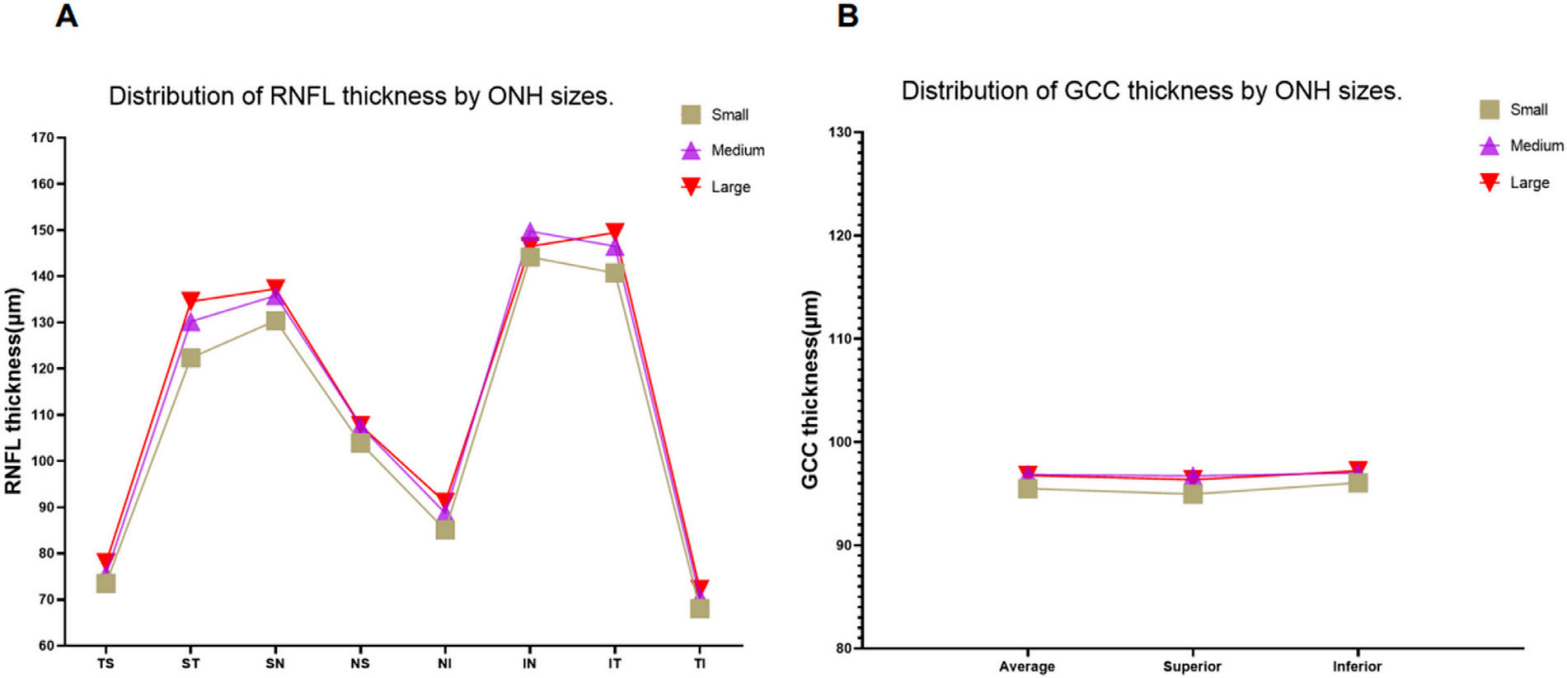
Distribution of RNFL and GCC thickness by ONH sizes. (A) The overall RNFL thickness distribution was the same for different types of ONH, and the thickness decreased from the inferior quadrant to the superior, nasal, and temporal quadrants. The RNFLs of eight segments were all thicker in large ONHs than in medium and small ONHs. (B) The overall GCC thickness distribution was the same for different types of ONH, and the thickness decreased from the inferior quadrant to the average and superior quadrants. However, there was no statistical difference between different ONH groups. IN, inferior nasal; IT, inferior temporal; NI, nasal inferior; NS, nasal superior; SN, superior nasal; ST, superior temporal; TI, temporal inferior; TS, temporal superior.

#### Linear Regression Analysis of Influencing Factors of RNFL and GCC Thickness

Univariate and multivariate linear regression analyses were used to estimate the independent associations between ocular (SER, BCVA, IOP, AL, disc area, cup area, VCDR, MCD) and systemic (age, sex, body mass index [BMI]) parameters and RNFL thickness (Table 3). In the univariate linear regression analysis, decreased RNFL thickness was significantly associated with older age (*P* < 0.001), male sex (*P* = 0.001), longer AL (*P* = 0.047), smaller disc area (*P* = 0.020), higher VCDR (*P* < 0.001), and larger MCD (*P* = 0.001) (Table 3). In the multivariate analysis, RNFL thickness was used as the dependent variable, and the significant (*P* < 0.05) variables in the univariate analysis were further included in the multivariate linear regression analysis (Table 3). We then excluded variables that were no longer significantly associated with RNFL thickness. In the multivariate analysis, decreased RNFL thickness was found to be significantly associated with older age (*P* = 0.003), male sex (*P* = 0.018), smaller disc area (*P* < 0.001), higher VCDR (*P* < 0.001), and larger MCD (*P* = 0.007) when age, sex, AL, disc area, VCDR, and MCD were assessed as independent variables.

**Table 3.** Univariate and Multivariate Analysis of the Relationship Between Demographic and Biochemical Characteristics and RNFL Thickness

	Univariate Analysis				Multivariate Analysis			
Variable	B	β	95% CI of B	*P*	B	β	95% CI of B	*P*
Sex	4.687	0.166	1.821, 7.552	**0.001**	3.403	0.121	0.581, 6.224	**0.018**
Age (y)	−0.396	−0.198	−0.598, −0.194	**<0.001**	−0.300	−0.150	−0.493, −0.106	**0.003**
BMI	0.180	0.055	−0.156, 0.516	0.293				
IOP (mm Hg)	−0.190	−0.032	−0.801, 0.421	0.541				
BCVA (logMAR)	−6.182	−0.073	−14.914, 2.551	0.165				
SER (diopter)	0.659	0.064	−0.406, 1.724	0.224				
AL (mm)	−1.563	−0.104	−3.103, −0.024	**0.047**	−1.254	−0.084	−2.752, 0.244	0.101
Disc area (mm^2^)	2.913	0.122	0.468, 5.358	**0.020**	5.442	0.228	2.962, 7.923	**<0.001**
Cup area (mm^2^)	−0.011	−0.019	−0.070, 0.048	0.717				
VCDR	−31.200	−0.212	−46.018, −16.382	**<0.001**	−36.294	−0.247	−51.611, −20.977	**<0.001**
MCD (mm)	−0.082	−0.180	−0.129, −0.036	**0.001**	−0.060	−0.132	−0.104, −0.016	**0.007**

Values with statistical significance are shown in boldface.

CI, confidence interval.

Univariate and multivariate linear regression analyses were used to estimate the independent associations between ocular (SER, BCVA, IOP, AL, disc area, cup area, VCDR, MCD) and systemic (age, sex, BMI) parameters and GCC thickness (Table 4). In univariate linear regression analysis, thinner GCC was significantly associated with older age (*P* = 0.001), male sex (*P* = 0.045), larger BCVA (*P* = 0.019), and higher VCDR (*P* = 0.001) (Table 4). In the multivariate analysis, GCC thickness was used as the dependent variable, and the significant (*P* < 0.05) variables in the univariate analysis were further included in the multivariate linear regression analysis (Table 4). We then excluded variables that were no longer significantly associated with GCC thickness. In the multivariate analysis, decreased GCC thickness was found to be significantly associated with older age (*P* = 0.018), larger BCVA (*P* = 0.023), and higher VCDR (*P* = 0.002) when age, sex, BCVA, and VCDR were assessed as independent variables.

**Table 4.** Univariate and Multivariate Analysis of the Relationship Between Demographic and Biochemical Characteristics and GCC Thickness

	Univariate Analysis				Multivariate Analysis			
Variable	B	β	95% CI of B	*P*	B	β	95% CI of B	*P*
Sex	1.704	0.105	0.035, 3.373	**0.045**	1.433	0.088	−0.237, 3.104	0.092
Age (y)	−0.204	−0.177	−0.321, −0.087	**0.001**	−0.144	−0.125	−0.264, −0.024	**0.018**
BMI	0.051	0.027	−0.143, 0.246	0.603				
IOP (mm Hg)	0.124	0.036	−0.229, 0.477	0.489				
BCVA (logMAR)	−6.021	−0.123	−11.040, −1.002	**0.019**	−5.903	−0.120	−10.974, −0.832	**0.023**
SER (diopter)	−0.077	−0.013	−0.693, 0.540	0.807				
AL (mm)	−0.263	−0.030	−1.157, 0.630	0.563				
Disc area (mm^2^)	0.093	0.007	−1.330, 1.515	0.898				
Cup area (mm^2^)	−0.002	−0.005	−0.036, 0.032	0.923				
VCDR	−14.658	−0.173	−23.285, −6.032	**0.001**	−13.418	−0.158	−21.977, −4.859	**0.002**
MCD (mm)	−0.006	−0.021	−0.033, 0.022	0.690				

Values with statistical significance are shown in boldface. CI, confidence interval.

## Discussion

Our results showed that RNFL thickness decreased from the inferior quadrant to the superior, nasal, and temporal quadrants, consistent with most previously reported studies.^24^ RNFL and GCC measurements have the same diagnostic value in evaluating glaucoma.^25^ After dividing all participants into three ONH size groups based on optic disc area, we found a significant difference in RNFL thickness (except for the nasal and inferior quadrants) according to OHN size, although there was no significant difference in GCC thickness between the groups (Table 2). Various factors may affect RNFL and GCC thickness evaluation. We further analyzed the influencing factors of RNFL and GCC thickness using multivariate regression analyses and found that sex, age, disc area, VCDR, and MCD were associated with RNFL thickness (Table 3), and age, BCVA, and VCDR were associated with GCC thickness (Table 4). Multivariate regression analysis also revealed a significant effect of ONH size on RFNL thickness measurements but not on GCC thickness measurements. In this study, the influence of ONH size on GCC thickness was not significant, consistent with the results reported by Rao et al.^20^ Compared with RNFL thickness, GCC thickness did not change with ONH size and had fewer influencing factors.

RNFL measurements are affected by many factors. A histologic study demonstrated that RNFL thickness decreased with increasing distance from the optic nerve margin.^26^ Therefore, the distance from the scan area to the edge of the optic nerve may be the primary influencing factor of ONH size on RNFL measurements. Eyes with different ONH sizes were scanned using a fixed-size area, which may have resulted in RNFL thickness measurements at different distances from the margin of the ONH. Kaushik et al.^27^ used the standard “fast” RNFL scan protocol and proportional 2.27 × disc scan protocol to measure peripapillary RNFL thickness and found that RNFL was significantly thinner using the proportional scanning protocol compared with the standard 3.4-mm protocol. Therefore, the fixed scan area is closer to the edge in the large ONHs than in the small ONHs, which may result in thicker RNFL measurements for the large ONHs. However, the main part of the GCC measurement involves the macula, which avoids the interference of abnormal ONH structures. Our results show that GCC thickness measurements were not affected by ONH size. Similarly, Vidas et al.^28^ showed that GCC parameters showed a slightly better glaucoma discriminating ability and were better predictors for the development of glaucoma than RNFL.

Compared to GCC, RNFL measurements have certain limitations in diagnosing glaucoma with different ONH sizes. In general, medium ONHs are the most common among the different ONH types, whereas large and small ONHs are less common. It is difficult to recognize glaucomatous optic nerve changes in large ONHs because of the large optic cup in both glaucomatous and physiologic large cup eyes; patients with small ONHs have smaller optic cups, and early glaucoma is more likely to be missed in such cases. RFNL is considered a useful method for assessing the structural loss of RGCs in glaucoma, although this method is affected by the size of the ONH and does not take into account the cell bodies and dendritic layers located in the ganglion cell layer (GCL) and inner plexiform layer (IPL), respectively.^9^^,^^29^^,^^30^ However, GCC measurements included changes in the three-layer structure of the RNFL, GCL, and IPL,^4^ and the GCC was thicker and included more information than RNFL measurements. A study of the prevalence and associated factors of segmentation errors in the peripapillary RNFL and macular GCC showed that a low signal strength index (SSI), large ONHs, and disease type were significantly correlated with RNFL segmentation failure, whereas SSI was the only baseline factor that was significantly associated with GCC segmentation failure,^31^ which is consistent with the results of the relationship between ONH size and RNFL and GCC thickness in our study. Therefore, in the diagnosis of early glaucoma with different ONH sizes, GCC can reflect retinal damage more accurately and stably than RNFL. Therefore, GCC may replace RNFL as an important indicator of glaucoma damage; however, further studies are needed to determine the effect of ONH size on the specificity and sensitivity of peripapillary RNFL and macular GCC.

Our study has several limitations. First, the participants included in this study were Chinese individuals aged ≥50 years; therefore, the results obtained do not apply to other ethnic or age groups. Second, patients with hypertension and diabetes were not excluded from the study. An older population in southern Italy showed that GCC thickness was inversely associated with hypertension.^32^ Correspondingly, RNFL thickness was reduced in patients diagnosed with diabetes.^18^ Therefore, hypertension and diabetes mellitus are potential risk factors for RNFL and GCC thickness reduction. Third, the excluded participants were significantly older than the included ones (*P* = 0.026), and age was significantly related to RNFL and GCC thickness^19^^,^^33^^,^^34^; the exclusion of participants may have influenced the current results. Fourth, the thickness of the RNFL and GCC was measured by Optovue OCT in this study, which may be different from that measured by other OCT imaging devices. Fifth, this study did not correct for the magnification effect caused by the axial length, which may cause bias in the results. Sixth, there were more female than male participants in this study, and this difference may also have affected the results.

In conclusion, our study showed that RNFL thickness was positively correlated with ONH size, whereas no significant association was observed between GCC thickness and ONH size. In the evaluation of early glaucoma damage, GCC may reflect retinal damage more accurately and reliably than RFNL because it is not influenced by ONH size, particularly in patients with large or small ONHs.
